# Prospective randomized controlled study on the effects of *Saccharomyces boulardii* CNCM I-745 and amoxicillin-clavulanate or the combination on the gut microbiota of healthy volunteers

**DOI:** 10.1080/19490976.2016.1267890

**Published:** 2016-12-14

**Authors:** Toufic A. Kabbani, Kumar Pallav, Scot E. Dowd, Javier Villafuerte-Galvez, Rohini R. Vanga, Natalia E. Castillo, Joshua Hansen, Melinda Dennis, Daniel A. Leffler, Ciarán P. Kelly

**Affiliations:** a Division of Gastroenterology, Department of Medicine, Beth Israel Deaconess Medical Center, Boston, MA, USA; b Harvard School of Public Health, Harvard University, Boston, MA, USA; c Molecular Research, Shallowater, TX, USA

**Keywords:** adult, antibiotic, antibiotic-associated diarrhea, *Clostridium difficile* infection, healthy, microbiota, probiotic, *Saccharomyces boulardii*

## Abstract

Probiotics are believed to be beneficial in maintaining a healthy gut microbiota whereas antibiotics are known to induce dysbiosis. This study aimed to examine the effects of the probiotic *Saccharomyces boulardii* CNCM I-745 (SB), the antibiotic Amoxicillin-Clavulanate (AC) and the combination on the microbiota and symptoms of healthy humans.

Healthy subjects were randomized to one of 4 study groups: SB for 14 days, AC for 7 days, SB plus AC, Control (no treatment). Participants gave stool samples and completed gastro-intestinal symptom questionnaires. Microbiota changes in stool specimens were analyzed using 16s rRNA gene pyrosequencing (bTEFAP).

Only one subject withdrew prematurely due to adverse events. Subjects treated by *S boulardii* + AC had fewer adverse events and tolerated the study regimen better than those receiving the AC alone. Control subjects had a stable microbiota throughout the study period. Significant microbiota changes were noted in the AC alone group during antibiotic treatment. AC associated changes included reduced prevalence of the genus *Roseburia* and increases in *Escherichia, Parabacteroides*, and *Enterobacter*. Microbiota alterations reverted toward baseline, but were not yet completely restored 2 weeks after antibiotherapy. No significant shifts in bacterial genera were noted in the SB alone group. Adding SB to AC led to less pronounced microbiota shifts including less overgrowth of *Escherichia* and to a reduction in antibiotic-associated diarrhea scores.

Antibiotic treatment is associated with marked microbiota changes with both reductions and increases in different genera. *S. boulardii* treatment can mitigate some antibiotic-induced microbiota changes (dysbiosis) and can also reduce antibiotic-associated diarrhea.

## Introduction

Interactions between intestinal flora and the human host play a major role in maintaining intestinal health as well as in the pathogenesis of several disorders including antibiotic-associated diarrhea (AAD) and Clostridium difficile infection (CDI).^1-5^ Gut microbes play crucial roles in salvaging energy, absorbing nutrients, enhancing trophic effects on the intestinal epithelium, mediating the development and maturation of the host immune system and protecting against colonization by pathogenic microbes including C. difficile.^1-8^

With emerging data about their proposed benefits in maintaining intestinal health, probiotics continue to gain popularity.^9-12^ Probiotics are active bacterial and yeast organisms that are well-tolerated and safe, and are available in different foods and dietary supplements.^9,11,12^ In sufficient doses, probiotics can provide significant health benefits. For example, the probiotic yeast Saccharomyces boulardii CNCM I-745 (SB) has been used to prevent and treat diarrheal diseases such as AAD for many decades.^9,10,13-15^ 8 of 10 randomized controlled trials indicated statistically significant protection from AAD in subjects taking SB in comparison to controls.^10^ A meta-analysis that pooled the results of SB trials for the prevention of AAD, demonstrated a relative risk of 0.47 (95% C.I. 0.35 – 0.63, p<0.001).^14^ Moreover, SB may also be beneficial for the prevention of recurrent CDI.^13,16^Some of the described mechanisms of action of SB include interference with bacterial adhesion, inactivation of toxins and other virulence factors, and enhancement of mucosal immune function.^9^

Conversely, antibiotics disrupt normal gut microorganisms and this dysbiosis impairs “colonization resistance” making the subject more vulnerable to opportunistic overgrowth within the microbiota.^3,7^ This increases the risk of CDI if exposure to toxigenic C. difficile occurs during or shortly after antibiotic use.^3,7,17^ Widespread use of antibiotics in the elderly, frail, hospital and nursing home patients place these already vulnerable populations at greater risk. Indeed, CDI is now the leading cause of nosocomial infectious diarrhea in the developed world with dramatic recent increases in incidence, death rates and healthcare costs.^3,7,17,18^

The aim of this study was to compare and contrast the effects of a probiotic (Saccharomyces boulardii CNCM I-745), an antibiotic (Amoxicillin-Clavulanate) and the combination on the intestinal microbiota of healthy humans. We also examined the effects of these interventions on gastro-intestinal symptoms in healthy subjects with a particular focus on AAD. Examining these effects can play a central role in understanding the dysbiosis induced by antibiotic treatment, its role in the pathophysiology of AAD (and potentially CDI) and the utility of SB in mitigating antibiotic-associated dysbiosis and/or AAD.

## Results

Among the 53 subjects enrolled in the study, 49 were considered for statistical analyses. At baseline, groups were comparable with regard to demographics, vital signs, and medical/treatments characteristics. Subjects had a mean age of 30 y [range 18 to 51] and 41% were males. They were all in good general health. One subject was excluded because of therapeutic antibiotic use. No other subject received a treatment in the exclusion listing (Table 3). Vitamin supplements and oral contraceptives were the most commonly used treatments. In treatment groups, 94.4% of subjects took at least 80% of the prescribed doses of study treatment.

### Gastrointestinal symptom rating scale (GSRS): Total score analysis

Among the 4 groups of subjects, the GSRS total score (mean ± SD) varied from 18.2 (±4.0) during the baseline period from Days −7 to 0, to 21.9 (±9.7) during the period from Days 0 to 7, 18.1 (±4.3) during the period from Days 7 to 14 and 17.5 (±4.5) during the period from Days 14 to 21 (Table 1a).

**Table 1a.** GSRS total scores by treatment group and study period.

	Group of randomization				
Gastrointestinal Symptom Rating Scale - Scores	Group 1 Sb (N = 13)	Group 2 AC (N = 12)	Group 3 Sb + AC (N = 12)	Group 4 Control (N = 12)	Total (N = 49)
Gastrointestinal symptoms total score D-7 to D0					
Missing	0	0	0	12	12
N	13	12	12	0	37
Mean ( ± SD)	18.7 ( ± 5.4)	18.7 ( ± 3.8)	17.3 ( ± 2.4)	NA ( ± NA)	18.2 ( ± 4.0)
Min-Max	[15.0;35.0]	[15.0;26.0]	[15.0;22.0]	[NA; NA]	[15.0;35.0]
Gastrointestinal symptoms total score D0 to D7					
Missing	0	0	0	0	0
N	13	12	12	12	49
Mean ( ± SD)	23.2 ( ± 8.8)	26.9 ( ± 14.2)	18.1 ( ± 3.0)	19.3 ( ± 8.1)	21.9 ( ± 9.7)
Min-Max	[15.0;44.0]	[15.0;59.0]	[15.0;25.0]	[15.0;43.0]	[15.0;59.0]
Gastrointestinal symptoms total score D7 to D14					
Missing	1	1	0	0	2
N	12	11	12	12	47
Mean ( ± SD)	19.3 ( ± 5.1)	20.3 ( ± 5.9)	16.8 ( ± 1.6)	16.4 ( ± 2.7)	18.1 ( ± 4.3)
Min-Max	[15.0;29.0]	[15.0;34.0]	[15.0;20.0]	[15.0;23.0]	[15.0;34.0]
Gastrointestinal symptoms total score D14 to D21					
Missing	1	1	0	0	2
N	12	11	12	12	47
Mean ( ± SD)	19.3 ( ± 5.7)	18.4 ( ± 5.8)	16.5 ( ± 2.9)	15.8 ( ± 2.3)	17.5 ( ± 4.5)
Min-Max	[15.0;33.0]	[15.0;34.0]	[15.0;25.0]	[15.0;23.0]	[15.0;34.0]

The higher value, i.e. a worsening of the gastrointestinal symptoms, was observed in the group of subjects treated by antibiotics alone (AC group) during the treatment period (Days 0 to 7), reaching 26.9 (±14.2) (Table 1a). This difference was statistically different between the GSRS total score for the AC group at Day 7 compared with Day 0 (increases: 8.25 (±2.74) [13.78; 2.72]; non-adjusted p-value = 0.0044). This significant worsening of GSRS total score compared with Day 0 was maintained during the week after the antibiotic treatment. The symptoms subsequently improved steadily with a drop of mean scores by 6.81 ( ± 2.54) points on Day 14 (p = 0.01) and 8.6 ( ± 2.61) points by Day 21 (p = 0.005) in comparison to Day 7 scores. At study completion, the mean GSRS score of the AC group was statistically similar to the group's baseline (Day 0) as well as the control group's score on Day 21. The differences in means were 1.6 ( ± 1.65) and 1.82 ( ± 1.85) (p = 1.0, and 0.7 respectively).

During the same treatment period there were no significant changes in the intra-group, total GSRS, scores for the SB alone group or for the SB+AC group.

The inter-group differences were also evaluated. At Day 0, the GSRS total scores of each group were comparable. At Day 7, the GSRS total score was significantly higher in the AC group compared with the SB+AC group (difference: 8.83 ( ± 3.84) [1.10; 16.57]; non-adjusted p-value = 0.0262).

### Gastrointestinal symptom rating scale (GSRS): Sub-scores analysis

**Figure 1.**
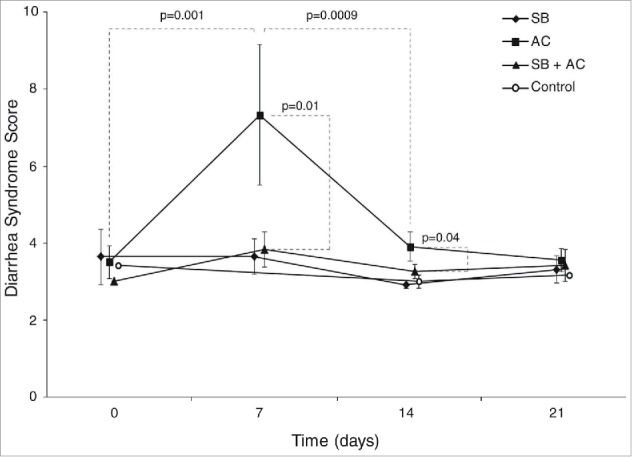
Diarrhea-GSRS sub-score from Gastrointestinal Symptoms Response Score (GSRS). Cumulative diarrhea- GSRS sub-score illustration for the 4 study groups at each study time-point: Control (n = 12), *Saccharomyces boulardii* CNCM I-745 (SB, n = 12), Amoxicillin-Clavulanate (AC, n = 12), *Saccharomyces boulardii* CNCM I-745 plus Amoxicillin-Clavulanate (SB + AC, n = 12).

After initiation of the treatments, all of the GSRS sub-scores from the SB+AC group were numerically lower than those from the SB alone group and from the AC group. The most substantial and statistically significant differences were related to the “diarrhea-GSRS sub-score” (comprising the sum of responses to GSRS questions 11, 12 and 13) (Fig. 1). The mean diarrhea-GSRS sub-score was statistically increased in the AC group on Day 7 compared with Day 0 (difference: 3.83 ± 1.10, p = 0.001).

On inter-group comparison, the mean diarrhea-GSRS sub-score for the AC group at day 7 was significantly elevated compared with the SB+AC group (difference: 3.92 ± 1.37 (p = 0.01) (Fig. 1). A significant difference was maintained at Day 14, one week after the end of antibiotic treatment (difference: 0.91 ± 0.30, p = 0.04) (Fig. 1).

All other symptom domains (reflux, abdominal pain, indigestion and constipation) were similar before, during and after treatment (intra-group analysis) with the exception of indigestion which was higher during the first week in the SB alone group (difference of means 2.15 ± 0.97, p = 0.03). However, this difference disappeared in the second week of treatment and did not recur. All symptom domains except diarrhea (reflux, abdominal pain, indigestion and constipation) were similar to the control group at corresponding study points (inter-group analysis).

### Daily stool log analysis

The number of stools per day (mean ± SD) over the 4 groups was stable across the study: 1.3 (±0.6) for the period D-7 to D-1, 1.3 (±0.7) for D0 to D9 and 1.3 (±0.5) for D10 to D21. As regards to the stool consistency, the percentage of unformed stools over the 4 groups increased from 11.3% [8.2%; 15.1%] at Day 0 to 24.5% [21.1%; 28.1%] at Day 10; then it decreased back to 15.2% [12.5%; 18.3%] at Day 21 (Table 1b). At the end therapy, the AC group had the highest percentage of unformed stools 33.1% [25.8%; 41.1%] whereas the SB+AC group had the lowest percentage of unformed stools 16.1% [10.5%; 23.1%]. The SB group and the control group presented with similar percentage of unformed stools, respectively 24.7% [18.4%; 31.9%] and 23.2% [16.7%; 30.7%]. These trends did not achieve statistical significance.

**Table 1b.** Daily stool log- Consistency of stools by group and by visit.

	Group of randomization				
N (%) [LCL;UCL]	Group 1 Sb (N = 13)	Group 2 AC (N = 12)	Group 3 Sb + AC (N = 12)	Group 4 Control (N = 12)	Total (N = 49)
**D-7 to D-1**					
✓ Formed	109 (90.1%)	100 (87.7%)	97 (88.2%)	NA (NA%)(a)	306 (88.7%)
	[83.3%;94.8%]	[80.3%;93.1%]	[80.6%;93.6%]	[NA%; NA%]	[84.9%;91.8%]
✓ Unformed	12 (9.9%)	14 (12.3%)	13 (11.8%)	NA (NA%)	39 (11.3%)
	[5.2%;16.7%]	[6.9%;19.7%]	[6.4%;19.4%]	[NA%; NA%]	[8.2%;15.1%]
**D0 to D9**					
✓ Formed	128 (75.3%)	105 (66.9%)	120 (83.9%)	116 (76.8%)	469 (75.5%)
	[68.1%;81.6%]	[58.9%;74.2%]	[76.9%;89.5%]	[69.3%;83.3%]	[71.9%;78.9%]
✓ Unformed	42 (24.7%)	52 (33.1%)	23 (16.1%)	35 (23.2%)	152 (24.5%)
	[18.4%;31.9%]	[25.8%;41.1%]	[10.5%;23.1%]	[16.7%;30.7%]	[21.1%;28.1%]
**D10 to D21**					
✓ Formed	121 (78.1%)	122 (87.1%)	131 (87.9%)	138 (86.3%)	512 (84.8%)
	[70.7%;84.3%]	[80.4%;92.2%]	[81.6%;92.7%]	[79.9%;91.2%]	[81.7%;87.5%]
✓ Unformed	34 (21.9%)	18 (12.9%)	18 (12.1%)	22 (13.8%)	92 (15.2%)
	[15.7%;29.3%]	[7.8%;19.6%]	[7.3%;18.4%]	[8.8%;20.1%]	[12.5%;18.3%]

*Note*. (a) For subjects of Group 4 (Control) the Visits 1 and 2 took place at the same day.

### Adverse events

In total, 14 of the 49 subjects (28.6%) reported adverse events (AEs) most of which were mild to moderate and related to gastrointestinal disorders including bloating, loose bowel movements and flatulence. 5 of the 13 subjects in the probiotic group (38.5%) reported AEs, whereas 6 of the 12 subjects in the antibiotic group (50%) reported AEs. Only 2 subjects in the SB+AC group (16.7%) reported AEs (Fisher's Exact Test p = 0.24). One subject in the control group (8.3%) reported AEs. Only one subject, in the probiotic group had AEs that led to his discontinuation from the study.

### Stool microbiota analysis results

**Figure 2.**
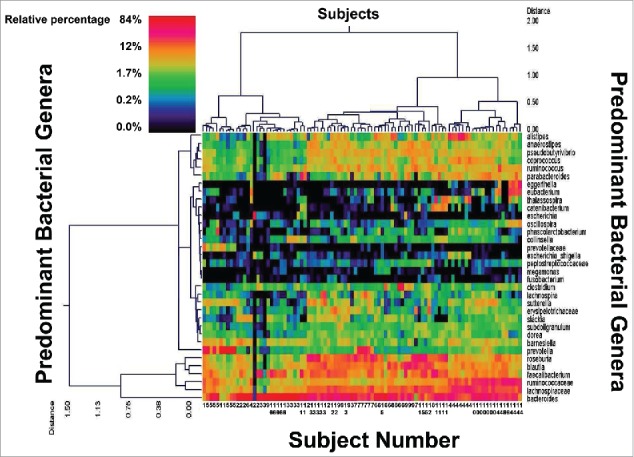
Dual Hierarchal Dendrogram of Subjects and their Predominant Fecal Bacterial Genera. Data for 16 of the 48 subjects who completed the study are shown coded by subject number. Control: Subjects 1, 8, 12 and 15; Saccharomyces *boulardii* CNCM I-745: Subjects 4, 7, 10 and 13; Amoxicillin-Clavulanate: Subjects 2, 5, 9 and 14; Saccharomyces *boulardii* CNCM I-745 plus Amoxicillin-Clavulanate: 3, 6, 11 and 16. Data for 7 samples are shown for each subject with the exception of the subjects from the control group who provided 3 samples each. The heat map represents the relative percentages of each bacterial genus. The predominant genera are represented along the right Y-axis. The legend for the heat map is provided in the upper left corner representing the relative percentages of each bacterial genus within each sample. Fecal samples with more similar microbial populations are closer together. The genera that are most abundant are *Faecalibacterium, Bacteroides, Roseburia*, and *Ruminococcus*.^49^ The figure illustrates that each subject maintains a relatively stable microbial population during the course of the study and that these populations are characteristic for that individual.

A total of 5,462,665 sequences were derived from 285 stool samples. After stringent quality sequence curation, a total of 3,036,204 sequences identified within the bacterial kingdom were utilized for final microbiota analyses with an average of 10,653 sequences per sample. A heat map of results from 4 subjects from each of the 4 study groups is presented in Figure 2. The main genera are shown along the right Y-axis. The most prevalent were Faecalibacterium, Bacteroides, Roseburia and Ruminococcus. Table 2 provides a summary of the 16 most prevalent bacterial genera within the study population according to treatment group and study phase (shown as mean percentage of total).

**Table 2.** Summary of the 16 most prevalent bacterial genera as determined by microbiota analysis according to treatment group and study phase (mean percentage of total).

		SB			AC			SB+AC		
Genera	Control	Before	During	After	Before	During	After	Before	During	After
*Bacteroides*	22.0	22.3	22.8	20.3	28.8	29.3	33.3	32.5	37.0	39.3
*Roseburia*	19.3	14.3	13.9	17.2	16.0	4.2	14.6	18.0	3.5	10.1
*Prevotella*	10.5	11.2	14.2	10.1	10.8	6.2	6.8	1.5	0.5	0.3
*Faecalibacterium*	6.9	6.4	5.5	6.7	5.9	9.5	7.8	5.7	10.5	8.0
*Ralstonia*	6.6	3.9	3.8	9.9	4.3	0.0	0.0	11.1	17.5	13.0
*Clostridium*	4.4	3.8	4.2	3.4	4.4	8.6	5.7	3.5	7.6	3.5
*Parabacteroides*	2.6	1.1	1.3	1.8	2.6	16.9	8.1	1.5	4.6	3.0
*Blautia*	4.5	4.2	3.6	3.7	4.7	2.9	5.4	4.2	2.4	3.7
*Ruminococcus*	4.3	5.1	4.8	4.2	4.0	2.4	4.5	4.9	2.1	2.9
*Coprococcus*	3.4	5.4	5.5	4.9	2.6	1.1	1.9	3.5	0.8	3.4
*Lachnospira*	2.5	3.7	4.6	1.8	2.6	0.8	2.0	1.9	0.1	0.2
*Oscillibacter*	1.1	2.1	2.3	3.5	1.2	1.2	1.3	1.2	0.4	0.6
*Sutterella*	0.4	2.9	1.9	1.4	1.6	0.6	1.2	0.8	0.6	0.7
*Alistipes*	2.2	1.3	1.5	1.0	0.9	0.5	0.6	1.5	1.0	0.9
*Eubacterium*	0.5	0.8	0.7	0.5	1.1	1.4	1.2	0.3	0.1	0.1
*Escherichia*	0.0	0.0	0.0	0.1	0.1	4.5	0.2	0.0	2.9	0.2

The heterogeneity of the human microbiota is well documented as is the relative stability of each individual's characteristic microbial architecture over time. Accordingly, each individual subjects' samples tended to group together when utilizing clustering of the microbial populations at the genus level, based upon Ward's Minimum Variance and Manhattan distance, as illustrated in Figure 2. These individual baseline microbiota tended to remain stable over time and to overshadow differences due to treatment effects.

The subjects' microbiota also tended to cluster into different groupings corresponding to the 3, so called, enterotypes classically described in the literature (Fig. 2). The 1^st^ cluster is characterized by the presence of Prevotella and lower amounts of Bacteroides (from subject 1 on the far left to the last sample of subject 11). The 2^nd^ cluster is characterized by high levels of Bacteroides (from subject 2 to the last sample of subject 11). The 3^rd^ cluster shows increased Ruminococcus (from subject 4 to the last sample of subject 14).

### Baseline bacterial genera prevalence differences related to age, gender and across treatment groups

We evaluated the core microbiota (derived from control and pretreatment samples) and compared the most abundant bacterial genera based upon gender, age or study group. We found that the prevalence of several genera were significantly different after accounting for repeated measurements. Females had significantly higher Alistipes (2.07% versus 0.84% in males), Oscillibacter (1.83% vs. 0.70%), Fusobacterium (1.16% vs. <0.01%), Anaerotruncus (0.56% vs. 0.18%), Cetobacterium (0.60% vs. <0.01%) and Oscillospira (0.11% vs. 0.04%). On the other hand, none of the bacterial genera identified was significantly more prevalent in males compared with females.

Overall, there were no notable differences related to age when studied as a continuous variable. Given the age distribution of the subjects, age was dichotomized as being less than 35 y or older. Younger subjects showed a significantly greater prevalence of Roseburia (18.42% vs. 11.76%), Oscillibacter (2.46% vs. 1.08%), Sutterella (2.79% vs. 0.96%), Megamonas (1.71% vs. 0.18%, Lactobacillus (1.02% vs. 0.06%) and Thalassospirira (0.47% vs. 0.07%).

When evaluating the baseline, pre-treatment samples we found significant differences in the prevalence of 3 genera (Table S1). Coprococcus abundance was significantly higher in the group assigned to later receive S. boulardii compared with the antibiotic group. Sutterella was significantly higher in the S. boulardii group compared with the S. boulardii plus antibiotic group and to the control group, and Catenibacterium was significantly higher in the control group compared with the S. boulardii group and to the antibiotic group.

Males and females were almost equally distributed among the study groups. In addition, the majority of the study participants were aged between 19 and 35 and the distribution by age was not statistically significant among the study groups (p = 0.8). Moreover, and to control for any potential effects attributed to age and gender distribution, we conducted multivariate analysis controlling for age and gender.

### Bacterial genera prevalence differences related to study treatments

Operational Taxonomic Unit (OTU) and Chao1 estimate analyses were performed on each of the samples. Both analyses showed that the antibiotic treated groups had significantly reduced bacterial diversity compared both to the control group's microbiota and to the S. boulardii only treatment group (Table S2 and S3).

**Figure 3.**
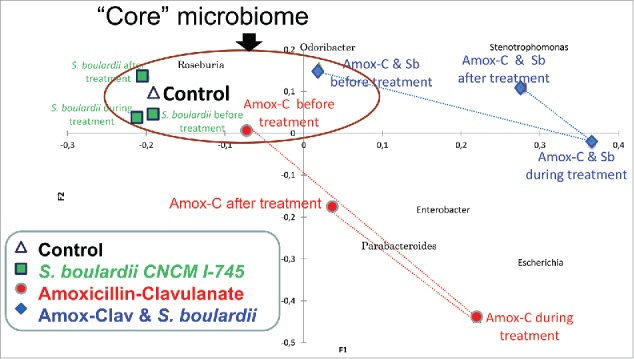
Biplot of Redundancy Analysis. Each point on the plot represents the total microbial assemblages of a treatment group at a single time point. The brown ellipse surrounds the baseline microbiota and represents the “core” microbiota for the study participants including the controls and each of the study groups prior to treatment. The antibiotic group (red circles) were shifted strongly away from the core microbiota both during and after treatment. The *S. boulardii* group (green squares) stayed within the core microbiota at all time points. The antibiotic plus *S. boulardii* group (blue diamonds) also showed a shift away from the core microbiota during treatment but this was less than for the antibiotic alone group. The bacterial genera that were significantly increased in prevalence during antibiotic treatment were *Escherichia, Parabacteroides*, and *Enterobacter* which are shown clustered primarily within the quadrant associated with the antibiotic group both during and after treatment. Although some differences in these groups were also associated with the antibiotic plus *S. boulardii* group the effects were most strongly related to the antibiotic alone group.

To further evaluate which treatments created notable bacterial population shifts we evaluated the microbial assemblage using analysis of similarity (ANOSIM, Table S4). Of the 3 treatments evaluated, only the S. boulardii alone group remained stable and did not have any significant shifts from its pre-treatment microbiota. The control group microbiota was not significantly different to the other groups before treatment. Hence the control group's samples and those taken from the other groups prior to any treatment can be evaluated together and combined to form a “core” microbiota (Fig. 3).

We also evaluated changes in the abundance of specific bacterial genera within study groups during and after study treatments. We used the one-way analysis of variance (ANOVA) method to compare multiple means of genera concentrations across the study groups (independent variables) whereas we used paired t-test to compare concentrations for dependent variables (inter-group changes corresponding to different study time points). Genera with significantly different prevalence between groups during treatment are presented in Table S5 while genera with significantly different prevalence between groups after treatment are presented in Table S6. Inter-group differences in prevalence were identified for 19 individual genera during the study treatments and for 22 individual genera after treatment.

To evaluate further the effects of study treatments, we took advantage of the total microbial assemblages within each treatment group. Our redundancy analysis biplot is shown in Figure 3. The core microbiota for the study participants encompassing the control and pre-treatment subjects' samples is outlined (brown ellipse, upper left quadrant). Samples obtained during and after antibiotic treatment shifted strongly away from the core microbiota (to lower, right quadrant). The bacterial genera that were most significantly increased during and after antibiotic treatment were Escherichia, Parabacteroides, and Enterobacter which are clustered primarily within the quadrant associated with antibiotic administration. Conversely, Roseburia prevalence was decreased by antibiotic treatment. Samples from the S. boulardii group remained completely within the core microbiota both during and after therapy. As for the group that combined S. boulardii administration with antibiotic treatment, there was also a notable, but less prominent and directionally distinct, shift away from the core microbiota that did not encroach closely upon the Escherichia-Parabacteroides-Enterobacter region. Differences in these genera were also statistically evident with the S. boulardii and antibiotic treatment group but the associations were not as strong as for the antibiotic alone group (Fig. 3). The antibiotic plus S. boulardii group showed treatment-related increases in Odoribacter and Stenotrophomonas that were not evident with the antibiotic or S. boulardii when given alone (Fig. 3, upper right quadrant).

### Correlations between antibiotic-associated diarrhea and prevalence of Escherichia

**Figure 4.**
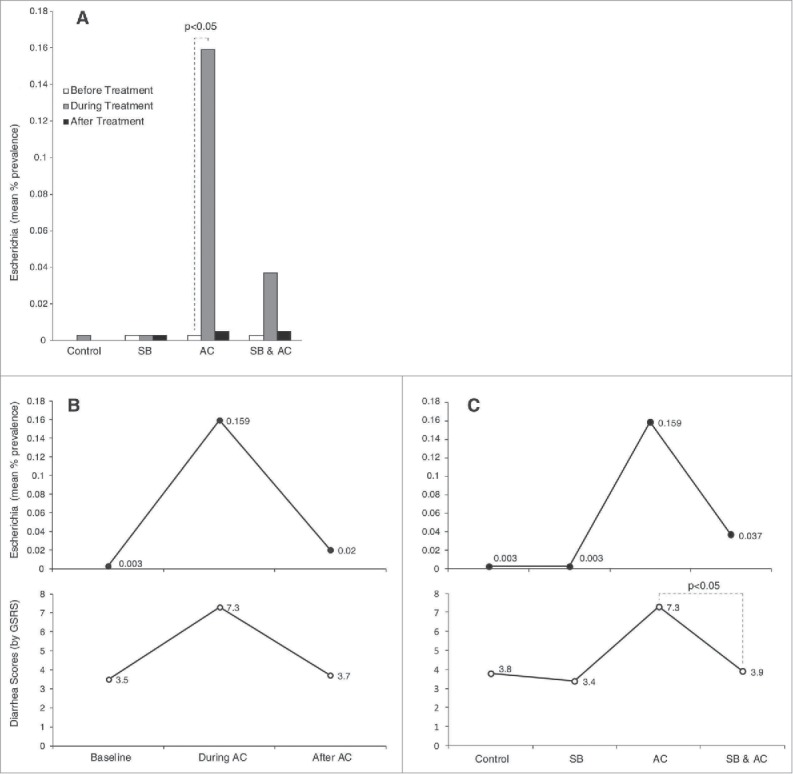
Correlations between diarrhea scores and *Escherichia* prevalence. (A) The mean percentage prevalence of *Escherichia* in stool samples are shown for each of the 4 study groups before, during and after treatments. (B, Upper panel) The mean percentage prevalence of *Escherichia* in stool samples are shown for the antibiotic group before, during and after treatment with Amoxicillin-Clavulanate. Lower panel: Corresponding symptom scores reported by subjects using the diarrhea domain of the Gastrointestinal Symptom Response Score (GSRS). (C, Upper panel) The mean percentage prevalence of *Escherichia* in stool samples are shown for the control group and for the *S. boulardii*, Amoxicillin-Clavulanate and combined treatment groups during treatment. Lower panel: Corresponding symptom scores reported by subjects using the diarrhea domain of the Gastrointestinal Symptom Response Score (GSRS).

Next we examined whether shifts in the concentrations of specific genera during and after therapy were associated with symptoms of antibiotic-associated diarrhea. Since significant microbiota changes were related to several genera including: Escherichia, Ralstonia and Parabacteroides, we conducted separate analysis to investigate whether there were any correlations between antibiotic-associated diarrhea and the prevalence of the genera that were most notable for microbiota changes. As illustrated in Figure 4A, the mean percentage prevalence of Escherichia in control and in S. boulardii treated subjects was very low (0.001% for each). Treatment with Amoxicillin-Clavulanate led to overgrowth of Escherichia increasing their prevalence by more than 100 fold (to 0.159%, p < 0.05). Conversely, when Amoxicillin-Clavulanate was administered in conjunction with S. boulardii the increase in Escherichia prevalence was substantially less (0.037%, not statistically significant). After treatment the prevalence of Escherichia fell to baseline in both the antibiotic alone and the antibiotic with S. boulardii groups (Fig. 4A).

As expected diarrhea scores (as measured by the diarrhea-GSRS sub-score) rose significantly during treatment with Amoxicillin-Clavulanate (p = 0.004) and returned to baseline after antibiotic treatment ended (Fig. 4B, lower panel). Overgrowth of Escherichia (as measured by mean percentage prevalence) correlated closely with symptoms of antibiotic-associated diarrhea (Fig. 4B, upper panel; R^2^ = 0.99, p < 0.001 by linear regression analysis). A correlation between Escherichia prevalence and diarrhea symptoms was also seen when comparing across groups (Fig. 4C). Most importantly, the blunting of Escherichia overgrowth that was observed when S. boulardii was administered together with Amoxicillin-Clavulanate mirrored a similar and significant reduction in antibiotic-associated diarrhea (Fig. 4C). The strong trend seen with Escherichia was not seen with Ralstonia and Parabacteroides and there did not appear to be a strong correlation between diarrhea symptoms and the relative abundances of these 2 genera.

As outlined above and as shown in Tables S2 and S3, antibiotic treatment was associated also with significant changes, either increases or decreases, in multiple other genera. Some, but not all, of these antibiotic-associated microbiota alterations were moderated significantly by S. boulardii co-treatment (Table S5). As an example, Amoxicillin-Clavulanate administration was associated with a substantial reduction in Ralstonia prevalence (from 6.57% to 0.019%, p < 0.05). S. boulardii co-treatment completely prevented this fall (17.5%, not significantly difference from control or from S. boulardii alone, see Table S5). Conversely, antibiotic treatment was associated with a substantial increase in Parabacteroides prevalence (from 2.64% to 16.89%, P < 0.05). S. boulardii co-treatment largely prevented this increase (4.59%, not significantly difference from control or from S. boulardii alone, see Table S5). Reductions in the prevalence of several other specific genera were seen during treatment with antibiotics and were not significantly altered by S. boulardii (e.g. Roseburia and Ruminococcus, see Table S5). Thus the moderating effect of S. boulardii on antibiotic-induced microbiota shifts appears to be selective rather than universal.

## Discussion

The human intestinal microbiota of a healthy individual is calculated to contain 100 trillion bacteria not including viruses, fungi, yeast, protists or archae.^2,4,6,8^ The richness and diversity of the human microbiota is influenced by many factors including mode of birth, diet, medications, age and debility.^2,8,19^ However, one of the most dramatic changes that can occur to the microbiota is that which results from antibiotic therapy. Millions of doses of antibiotics are administered in the US and worldwide each year.^20^ Antibiotics are associated with many side effects, among which AAD is the most common.^21,22^ Moreover, the incidence, morbidity and mortality associated with CDI have been steadily rising over the last decade and are expected to continue to rise given the emergence of hyper-virulent strains of C difficile and the growing use of antibiotics.^3,7,18^ In the lights of these hazards, it has become common practice for patients to consume foods rich in probiotics and/or over-the-counter probiotic supplements when using antibiotics.^5^ However, despite this common practice and the significant impact of AAD and CDI on public health, there are few studies that document, whether and/or how, the use of probiotics impacts gastrointestinal (GI) symptoms and microbial composition of healthy subjects using antibiotics. In this study we aimed to explore the utility of a probiotic (Saccharomyces boulardii CNCM I-745) that is commonly used to reduce or prevent AAD and determine if it prevents or minimizes gut-related side effects associated with a commonly used antibiotic, Amoxicillin-Clavulanate. Moreover, we examined the effects of antibiotics, probiotics and their combination on the composition of healthy subjects' microbiota.

Our study data demonstrate several important findings. The most marked changes to the intestinal microbiota were clearly those associated with antibiotic use (Fig. 3). These changes showed only partial reversal 14 d after antibiotic administration had ended. In this study, S. boulardii alone had no evident substantial effect on the intestinal microbiota of healthy individuals. Other studies have evaluated the effects of probiotics on the composition and diversity of the intestinal microbiota, both in patients and healthy individuals.^23^ For S. boulardii CNCM I-745, in particular, studies using conventional culture-based methods or FISH have shown that it does not substantially modify microflora composition.^24,25^

Although S. boulardii alone does not alter the microbiota, in this study the combination of S. boulardii with Amoxicillin-Clavulanate modified the microbiota changes caused by an antibiotic. Modulation of the intestinal microbiota may be one of the main beneficial effects of probiotics. These modulatory effects seem to be selective rather than universal since S boulardii mitigated shifts in the relativeabundances ofRalstonia and Parabacteroides seen with Amoxicillin-Clavulanate use but did not seem to have significant influences on other genera such as Roseburia and Ruminococcus. The impact of S. boulardii CNCM I-745 following disruption of microbiota by antibiotics was investigated in several animal models. The results suggested that S. boulardii CNCM I-745 allows a quicker return to basal level of fecal microbiota population.^26,27^ This is the first study to report these findings using 16s sequencing. However, our findings are supported by earlier reports using other techniques. The effect of S. boulardii CNCM I-745 was confirmed in a clinical study that investigated the fecal microbiota (investigated by FISH and microscopic biostructure) in 20 healthy volunteers and 20 patients with chronic idiopathic diarrhea. The microbiota changes and clinical symptoms in those with chronic idiopathic diarrhea were improved with S. boulardii CNCM I-745 therapy.^25^ More recently, a clinical study was conducted in 60 women treated for bacterial vaginosis with ciprofloxacin and metronidazole. Fecal microbiota composition was analyzed by the FISH method. The authors concluded that S. boulardii CNCM I-745 was effective in preventing or reducing antibiotic-associated changes in colonic microbiota, when given concomitant with or subsequent to antibiotic therapy.^28^

Our study also confirmed the findings reported by multiple studies in the past which associated the use of antibiotics with higher incidence of diarrhea and/or loose bowel movements.^21,22^ 6 of the 12 subjects in the AC group, who had formed bowel movements prior to AC ingestion, had more than 3 loose bowel movements during antibiotic use. Moreover, subjects in the AC group reported statistically significantly higher diarrhea scores compared with the control, probiotic and combination regimen groups (Fig. 1).

5 of the 12 subjects who were in the SB group reported AEs. Nonetheless, most of the reported symptoms were mild and overall, well tolerated. Only one subject voluntarily withdrew from the study due to an adverse event. While one might expect that the co-ingestion of 2 agents with potential side effects (AC and SB) would increase the overall reported symptoms compared with each separately, adding SB to AC led to a reduction in diarrhea associated with AC without worsening other GI syndromes. On the contrary, indigestion reported within the first week of probiotic use in the SB group, seemed to be less of a problem when SB was co-administered with AC. Finally, only 16.7% of the combination AC plus SB group subjects reported symptoms compared with 50% of the AC alone group subjects. Probiotics are often administered during a course of antibiotics, and our results support this practice.

Another interesting observation of our study was that the microbiota profiles associated with our study appeared to form clusters as seen in Figure 2. This could be consistent with the findings previously reported by Arumugam who suggested that individuals may be separated into one of 3 major enterotypes. Indeed the MetaHit consortia (http://www.metahit.eu/), has classified gut microbiota based on the most abundant genera into enterotypes^8^ where Enterotype 1 is Bacteroides dominant, Enterotype 2 is Prevotella dominant and Enterotype 3 is Ruminococcus dominant genera. These enterotypes appear to be independant of gender, age and ethnicity, but do depend upon the long-term diet of individuals.^29,30^ Based on this definition, our 1^st^ cluster is closer to Enterotype 2, and our 2^nd^ and 3^rd^ clusters are closer to Enterotype 1 and 3 respectively. However we didn't identify clear separation between these groups and a gradient of Bacteroides could be observed across our 3 clusters. The generalizability of the enterotype clustering has been questioned^31^ and has generated controversy in the scientific community. It is not clear if enterotypes are distinct entities or whether it is better to define gut microbiota as a continuum. This concept is supported by HMP consortia (http://hmpdacc.org/) which view gut microbiota as a gradient of species known as entero-gradient.^31,32^ With this concept of entero-gradient, the observed associations of so called enterotype occur at the extremes.^31,33-36^ While some authors failed to identify enterotype clusters, especially concerning the Ruminococcus enterotype,^37^ other groups claim that Bacteroides and Prevotella do not exist in equal proportion in the gut,^35^ and seem to not co-exist in the same gut environment. Bacteroides and Prevotella compete for the same niche hence the concept of entero-gradient may offer a better description of bacterial communities than so-called enterotypes.^34^

Age, gender, and ethnicity did not appear to correlate with our cluster category. Despite the fact that using antibiotics caused transient microbiota shifts, intra-personal variations in almost all the study subjects were significantly less than inter-personal variations. This is probably due to complex interactions of diet, life-style and genetic/host factors that are crucial for maintaining a relatively stable, individualized and “healthy” microbiota profile over time.^2,8,19,38,39^ Another interesting observation was the tendency for the microbiota to revert toward the baseline status in these healthy individuals at the end of antibiotic use.

Interestingly, clinical recovery (resolution of side effects and symptoms) paralleled the recovery of the baseline microbiota. This fact helps to better understand the pathophysiology of AAD and CDI. Failure to restore the same “healthy” microbiota is well documented in recurrent CDI and constitutes the scientific mechanism upon which fecal microbial transplantation (FMT) is based.^40^ In the future, it will be important to determine whether a certain “enterotype” group is associated with higher risk of developing gut diseases such as AAD, CDI, irritable bowel disease, and inflammatory bowel diseases, or non-GI diseases that are impacted by gut microbial composition such as metabolic or auto-immune disorders. Equally important would be to explore microbiota variations before and after diagnosis and/or effective treatment of such disorders.

Another important observation was the evident overgrowth of Escherichia in the stools of subjects during AC use. Furthermore, we found that the higher the mean concentrations of Escherichia, the worse were the observed diarrhea scores. Moreover, the improvement and resolution of diarrhea paralleled the decline in Escherichia prevalence over the 2 weeks following cessation of antibiotics. Such strong correlations were not seen with other genera. These findings are consistent with the hypothesis that microbiota alterations, such as an overgrowth of Escherichia, underlie the mechanisms that lead to AAD. Proposed mechanisms include the direct effects of small molecules related to Escherichia in affecting colonic cells absorptive capacity. Other mechanisms could be more complex and related to “neutralizing” the beneficial effects of protective strains such as Parabacteroides through bacterio-bacterial interactions. Adding SB to AC minimized the observed rises in mean Escherichia concentrations and was simultaneously associated with lower diarrhea scores compared with the AC alone group (Fig. 4). Moreover, when Escherichia concentrations were lowest (control group), diarrheal score were lowest. These initial findings are worth further investigation with additional animal and human studies.

While our study reached several important and novel findings, we do note some limitations. First, we studied the effects of a specific probiotic (SB) and antibiotic (AC) on clinical symptoms and the microbiota of healthy volunteers. Therefore, our findings are not necessarily generalizable to other populations (e.g., those with illnesses) or to other antibiotics or probiotics. While the study subjects' compliance rates with drug administration and sample collections were very high, this study was conducted in an outpatient setting. Therefore, true compliance with medication administration is not guaranteed. Our study was not blinded. Therefore, some of the improved symptoms attributed to use of probiotics can be due to a placebo effect. Finally, our analyses involved frozen stool samples; hence inconsistencies in the handling of stool samples, outside of the study protocol, cannot be excluded.

In conclusion, we found that antibiotic therapy with Amoxicillin-Clavulanate led to substantial alterations of the microbiota of healthy subjects. This included an overgrowth of Escherichia and was accompanied by antibiotic-associated diarrhea. Amoxicillin-Clavulanate was also associated with reduced prevalence of the genus Roseburia and increases in Parabacteroides and Enterobacter in addition to Escherichia. Microbiota alterations reverted toward baseline but were still not completely resolved 2 weeks after completion of antibiotic administration. The probiotic S. boulardii CNCM I-745 given alone did not appreciably alter microbiota composition or symptoms. However, when S. boulardii was administered together with Amoxicillin-Clavulanate, microbiota changes, including Escherichia overgrowth, were lessened and antibiotic-associated diarrhea was prevented. Given the ability of S. boulardii, when administered with Amoxicillin-Clavulanate, to reduce Escherichia overgrowth and to induce significant increases or decreases in multiple other genera, future studies are warranted to explore the link between specific microbiota components and their metabolic products including small molecules and symptoms of antibiotic associated diarrhea.

## Subjects and methods

### Study design

**Figure 5.**
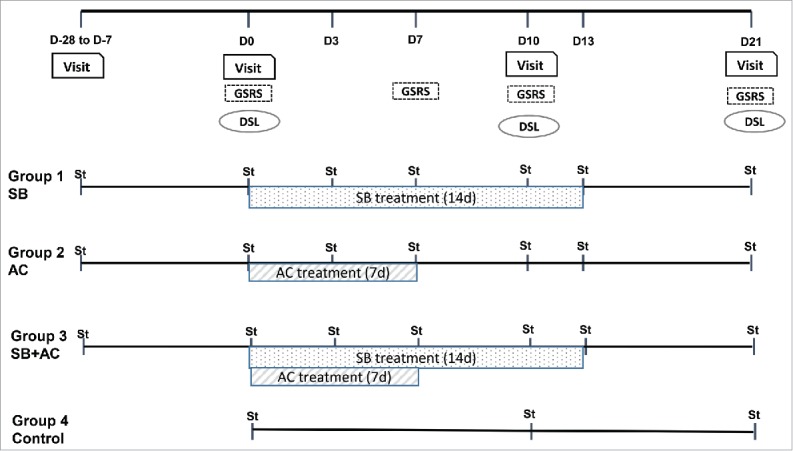
Study design. Summary outline of the clinical study design, study groups, endpoints and stool sample collection times. D: day; St: stool samples

This was a single-center, open-label, randomized controlled trial in healthy volunteers. All study visits were held at the Harvard Catalyst, Clinical and Translational Science Center at Beth Israel Deaconess Medical Center, in Boston, Massachusetts. The Institutional Review Board at Beth Israel Deaconess Medical Center approved this study. Healthy volunteers were informed about the risks and requirements of participating in the study and gave their written, informed consent before any study procedure was done. Included subjects had to be aged 18 to 65 (both genders), to have a good general health status, and no immunocompromise or hypersensitivity to yeasts, penicillins or cephalosporins (Table 3). They were randomized using a computer generated randomization sequence, to receive either Saccharomyces boulardii (SB) CNCM I-745 (syn. CBS 5926) (Florastor®, Biocodex Inc.) for 14 d (500 mg twice daily), or Amoxicillin-Clavulanate (AC) for 7 d (875/125 mg, twice daily at least 1 hour before meals), or the combination (SB+AC, same regimen as for each component given alone), or no treatment (control group). The target number of study participants to complete the protocol was 48 subjects (12 subjects per group); dropouts were replaced. The study design is illustrated in Figure 5.

**Table 3.** Inclusion and exclusion criteria for healthy volunteers.

**Inclusion criteria**
• Age 18 to 65 y (male or female)
• Good general health
• Able to comply with study requirements and to provide informed consent
• For women of childbearing potential
• A negative urine pregnancy test immediately prior to starting the study treatment
• Agreement to comply with approved methods of contraception during the period of active study treatment (not required during the follow-up)
**Exclusion criteria**
• History of organ transplantation
• Known chronic or recurrent systemic disorder associated with immunocompromise
• A history of allergy or hypersensitivity to *Saccharomyces boulardii*, brewer's or baker's yeast, Amoxicillin-Clavulanate, amoxicillin or other penicillins, or cephalosporins
• History of any severe allergic reaction (requiring hospital admission and/or the administration of parenteral medication or associated with dyspnea, wheezing, hypotension, loss of consciousness).
• Oral or systemic antibacterial therapy during the 3 months prior to study enrollment
• New prescription medications during the 4 weeks prior to study enrollment
• Prescription, over-the-counter medications or supplements that are known to alter gut function or microflora (i.e., acid anti-secretory drugs, probiotics) during the 4 weeks prior to study enrollment
• Active gastrointestinal disease
• Patient with a central venous catheter
• Patients taking antifungals or laxatives within 14 d of enrollment
• Prior gastrointestinal surgery (apart from appendectomy or cholecystectomy)
• History of chronic constipation with passage of fewer than 3 bowel movements per week on average
• History of chronic or recurrent diarrhea with spontaneous unformed bowel movements equivalent to or more often than 3 times daily
• History of CDI
• Any condition or personal circumstance that, in the opinion of the investigator, renders the subject unlikely or unable to comply with the full study protocol.
• Current smoker

### Study participants

53 subjects were enrolled between 07 May 2012 and 28 January 2013, of which 5 prematurely withdrew. One subject met an exclusion criterion (use of antibiotics), and 3 declined to participate after signing the consent form due to consent withdrawn, difficulties to commute to the site or no social security affiliation. These 4 subjects did not start any study regimen and were excluded from the Full Analysis Set. One additional subject from the SB group withdrew from the study after 5 d of treatment due to adverse events (bloating, loose bowel movements, regurgitation, and flatulence). This subject was included in the FAS population.

Study participants were asked to avoid throughout the study all probiotic supplementation, antacids, anti-diarrheal or anti-constipation medications, antifungals, additional antibiotics, and consumption of yogurt or fermented foods containing live yeast (see Table S7 for foods to be avoided). They were instructed to avoid any other changes to their usual diet. Compliance to study treatment and dietary restrictions was assessed and adverse events were recorded at each study visit (days 0, 10, and 21).

### Endpoints

The main endpoint was the change from baseline in the composition of the gut microbiota. For subjects receiving active treatment (SB, AC or SB+AC), fecal microbiota composition was examined on 7 occasions (Fig. 5): prior to (screening and day 0), during (days 3, 7, 10, 13) and after (day 21) treatment. 3 stool samples were collected from the control group (days 0, 10 and 21).

Other endpoints were Gastrointestinal Symptom Rating Scale (GSRS) scores and bowel movements characteristics recorded in a Daily Stool Log (frequency, consistency). The GSRS is a validated gastrointestinal symptom questionnaire consisting of 15 questions that address 5 main gastrointestinal syndromes: abdominal pain, constipation, diarrhea, indigestion and reflux (Table S8). Subjects were asked to score their symptoms experienced during the past week from 1 (no symptom) to 7 (very severe symptom) on days 0 (day preceding first study drug intake), 7, 14 and 21. Consistency of stools was evaluated in the Daily Stool Log as “formed” (solid and maintaining its shape after being passed) or “unformed” (watery or loose, and not maintaining its shape but instead taking on the shape of its container).

### Stool samples

Of the 288 requested stool samples (36 subjects with 7 samples plus 12 controls with 3 samples), 286 (99.3%) were returned in compliance with the study protocol. 2 subjects failed to supply one of the 7 requested samples: one subject did not have a bowel movement within that period (SB group) and one lost the sample on route to the study visit (AC group). As one additional sample could not be analyzed for technical reasons, 285 samples were included in the microbiota analysis.

### Fecal analysis methodology

DNA was extracted from fecal samples as has been described previously.^41-46^ Briefly, the microbiome profile was described using Tag-Encoded FLX Amplicon Pyrosequencing (bTEFAP®),^42^ targeting 16S rRNA gene V1-V3 region,^47^ utilizing Roche 454 FLX titanium instruments. The Q25 sequence data was processed using a proprietary analysis pipeline (MR DNA, Shallowater, TX). Briefly sequences were depleted of barcodes and primers. Short sequences < 200bp, sequences with ambiguous base calls, and sequences with homopolymer runs exceeding 6bp were sequentially removed. Sequences were then denoised and chimeras were removed. Operational taxonomic units (OTUs) were defined after removal of singleton sequences, clustering at 3% divergence (97% similarity).^41-46^ OTUs were taxonomically classified using BLASTn against a curated GreenGenes/RDP/NCBI derived database.^48^

Statistical analysis was performed using a variety of computer packages including XLstat, NCSS 2007, “R” and NCSS 2010. α and β diversity analyses were conducted as described previously.^41-46^ α diversity (number of different bacterial species) was determined by the number of operational taxonomic units (OTU) identified in a sample. β diversity (an analysis of bacterial community structure) was evaluated using phylogenetic trees, without regard for taxonomy. The “Core Microbiome” was defined as the grouping of baseline (control and pre-treatment) microbial community structures of the study subjects. A principal coordinate analysis was performed to allow for visualization of 10 separate jackknife iterative comparisons. The multidimensional space was then described within the 3 primary vectors. Data were evaluated in a multivariate manner to determine the effects of individuals, age, and gender. Significance reported for any analysis was defined as p<0.05.

### Clinical data analyses

Statistical analyses of relationships between changes in the microbiota and symptoms as reported by subjects (through the GSRS and stool logs) was performed using SPSS (for Windows, Rel. 13.0. 2004. Chicago. SPSS Inc). Outcomes were assessed using ANOVA, or Chi-square test with Yates correction for comparison of 2 or more means or proportions. The Benjamini-Hochberg procedure, that accounts for false discovery rate with more precision in comparison to the Bonferroni correction, was used to calculate the adjusted p value when comparing outcomes across different study groups (inter-group analysis). Linear regression analysis was used to explore correlations between bacterial concentrations and diarrhea scores. Significance reported for any analysis is defined as p<0.05.

The microbiota analysis (main objective) included subjects who completed the study according to the protocol and provided adequate series of samples. All other statistical analyses were performed on the Full Analysis Set (FAS) defined as all subjects who received at least one dose of study treatment or were included in the control group, and who provided at least one stool sample. It was identical to the Safety Set. The safety analysis was descriptive.

## Supplementary Material

KGMI_A_1267890_Supplemental.docx

## Abbreviations

AAD: antibiotic-associated diarrhea
AC: Amoxicillin-Clavulanate
AEs: Adverse events
BMI: Body Mass Index
CDI: *Clostridium difficile* infection
SB: *Saccharomyces boulardii* CNCM I-745
