# Supplementary material for: Prospective randomized controlled study on the effects of Saccharomyces boulardii CNCM I-745 and amoxicillin-clavulanate or the combination on the gut microbiota of healthy volunteers
Source: Gut Microbes. 2016 Dec 14;8(1):17–32. doi: 10.1080/19490976.2016.1267890 (PMC5341914; doi:10.1080/19490976.2016.1267890)
Supplement: KGMI_A_1267890_Supplemental.docx [file kgmi-08-01-1267890-s001.docx]

**Table S1: Genera which are significantly different in prevalence between the 4 groups prior to treatment.** Data are shown as mean percentage of total bacterial numbers. Groups that share a common letter are not significantly different from one another while groups that do not share a letter (A or B) are significantly different (P<0.05 for each). SB = *Saccharomyces boulardii* CNCM I-745, AC = Amoxicillin-Clavulanate.

| **Category** | ***Coprococcus*** | **Groups** | |
| --- | --- | --- | --- |
| SB | 5.375 | A |  |
| SB plus AC | 3.457 | A | B |
| Control | 3.351 | A | B |
| AC | 2.640 |  | B |
|  |  |  |  |
| **Category** | ***Sutterella*** | **Groups** | |
| SB | 2.895 | A |  |
| AC | 1.589 | A | B |
| SB plus AC | 0.769 |  | B |
| Control | 0.435 |  | B |
|  |  |  |  |
| **Category** | ***Catenibacterium*** | **Groups** | |
| Control | 1.818 | A |  |
| SB plus AC | 0.575 | A | B |
| SB | 0.001 |  | B |
| AC | 0.001 |  | B |

**TableS2:** **Operational Taxonomic Unit Analysis**

Observed species analysis showing a significant difference between the AC groups (which had significantly reduced bacterial diversity) compared to the core microbiome and the *S boulardii* only group. Groups that share a common letter are not significantly different from one another while groups that do not share a letter (A, B or C) are significantly different (P<0.05 for each). SB = *Saccharomyces boulardii* CNCM I-745, AC = Amoxicillin-Clavulanate, Core Microbiome = combination of all control and pre-treatment samples.

| **Variable** | **Mean** | **Std. deviation** | **Sum of ranks** | **Mean of ranks** | **Groups** | | |
| --- | --- | --- | --- | --- | --- | --- | --- |
| **SB & AC during** | 449.8 | 210.1 | 1589.0 | 72.2 | A |  |  |
| **SB & AC after** | 509.9 | 251.9 | 3056.0 | 92.6 | A | B |  |
| **AC during** | 461.1 | 228.2 | 1615.0 | 76.9 | A |  |  |
| **AC after** | 668.8 | 188.4 | 4431.5 | 134.3 |  | B | C |
| **Core Microbiome** | 730.9 | 240.4 | 17196.5 | 160.7 |  |  | C |
| **SB during** | 776.7 | 167.6 | 4199.0 | 175.0 |  |  | C |
| **SB after** | 749.1 | 239.0 | 5863.0 | 167.5 |  |  | C |

**Table S3:**  **Chao1 estimates**

Chao1 estimate analysis showed a significant difference between the AC groups (which had significantly reduced bacterial diversity) compared to the core microbiome and the *S boulardii* only group. Groups that share a common letter are not significantly different from one another while groups that do not share a letter (A, B or C) are significantly different (P<0.05 for each). SB = *Saccharomyces boulardii* CNCM I-745, AC = Amoxicillin-Clavulanate, Core Microbiome = combination of all control and pre-treatment samples.

| **Variable** | **Mean** | **Std. deviation** | **Sum of ranks** | **Mean of ranks** | **Groups** | | |
| --- | --- | --- | --- | --- | --- | --- | --- |
| **SB & AC during** | 794.2 | 391.5 | 1600.0 | 72.7 | A |  |  |
| **SB & AC after** | 942.5 | 525.5 | 1682.0 | 80.1 | A | B |  |
| **Core Microbiome** | 1342.4 | 495.1 | 3224.0 | 97.7 | A | B |  |
| **AC during** | 846.1 | 420.6 | 4357.0 | 132.0 |  | B | C |
| **AC after** | 1195.3 | 352.9 | 16886.0 | 157.8 |  |  | C |
| **SB during** | 1462.7 | 356.7 | 5890.0 | 168.3 |  |  | C |
| **SB after** | 1408.309 | 527.285 | 4311.000 | 179.625 |  |  | C |

## **Table S4: Analysis of similarity (ANOSIM) evaluation of taxonomy.**

P values for intergroup comparisons are shown. Significant differences between groups are presented in bold font. SB = *Saccharomyces boulardii* CNCM I-745, AC = Amoxicillin-Clavulanate

|  | **SB & AC during** | **SB & AC after** | **SB & AC before** | **AC during** | **AC after** | **AC before** | **Control** | **SB during** | **SB after** | **SB before** |
| --- | --- | --- | --- | --- | --- | --- | --- | --- | --- | --- |
| **SB & AC during** |  | 0.105 | **0.01** | 0.07 | **0.002** | **0.008** | **0.001** | **0.000** | **0.001** | **0.000** |
| **SB & AC after** |  |  | 0.6 | **0.006** | **0.02** | 0.1 | **0.002** | **0.004** | **0.001** | **0.01** |
| **SB & AC before** |  |  |  | **0.001** | 0.4 | 0.7 | 0.6 | **0.02** | 0.4 | 0.04 |
| **AC during** |  |  |  |  | **0.01** | **0.02** | **0.003** | **0.001** | **0.001** | **0.000** |
| **AC after** |  |  |  |  |  | 0.5 | 0.1 | 0.1 | **0.01** | 0.2 |
| **AC before** |  |  |  |  |  |  | 0.5 | 0.1 | 0.3 | 0.2 |
| **Control** |  |  |  |  |  |  |  | 0.7 | 0.8 | 0.9 |
| **SB during** |  |  |  |  |  |  |  |  | 0.8 | 0.9 |
| **SB after** |  |  |  |  |  |  |  |  |  | 0.9 |
| **SB before** |  |  |  |  |  |  |  |  |  |  |

**Table S5: Genera which are significantly different between groups during treatments.** Groups that share a common letter are not significantly different while groups that do not share a letter (A or B or C) are significantly different (P<0.05 for each). SB = *Saccharomyces boulardii* CNCM I-745, AC = Amoxicillin-Clavulanate.

| **Category** | ***Roseburia*** | **Groups** | |  |  |
| --- | --- | --- | --- | --- | --- |
| Control | 19.333 | A |  |  |  |
| SB | 13.900 | A |  |  |  |
| AC | 4.167 |  | B |  |  |
| SB plus AC | 3.493 |  | B |  |  |
|  |  |  |  |  |  |
| **Category** | ***Ralstonia*** | **Groups** | |  |  |
| SB plus AC | 17.529 | A |  |  |  |
| Control | 6.570 | A | B |  |  |
| SB | 3.842 | A | B |  |  |
| AC | 0.019 |  | B |  |  |
|  |  |  |  |  |  |
| **Category** | ***Parabacteroides*** | **Groups** | |  |  |
| AC | 16.892 | A |  |  |  |
| SB plus AC | 4.587 |  | B |  |  |
| Control | 2.638 |  | B |  |  |
| SB | 1.335 |  | B |  |  |
|  |  |  |  |  |  |
|  |  |  |  |  |  |
| **Category** | **Ruminococcus** | **Groups** | |  |  |
| SB | 4.752 | A |  |  |  |
| Control | 4.341 | A |  |  |  |
| AC | 2.366 |  | B |  |  |
| SB plus AC | 2.099 |  | B |  |  |
|  |  |  |  |  |  |
| **Category** | ***Coprococcus*** | **Groups** | | |  |
| SB | 5.495 | A |  |  |  |
| Control | 3.351 |  | B |  |  |
| AC | 1.087 |  |  | C |  |
| SB plus AC | 0.761 |  |  | C |  |
|  |  |  |  |  |  |
| **Category** | ***Lachnospira*** | **Groups** | |  |  |
| SB | 4.557 | A |  |  |  |
| Control | 2.459 | A | B |  |  |
| AC | 0.832 |  | B |  |  |
| SB plus AC | 0.104 |  | B |  |  |
|  |  |  |  |  |  |
| **Category** | ***Alistipes*** | **Groups** | |  |  |
| Control | 2.238 | A |  |  |  |
| SB | 1.495 | A | B |  |  |
| SB plus AC | 0.964 | A | B |  |  |
| AC | 0.510 |  | B |  |  |
|  |  |  |  |  |  |
|  |  |  |  |  |  |
| **Category** | ***Catenibacterium*** | **Groups** | |  |  |
| Control | 1.818 | A |  |  |  |
| SB plus AC | 0.240 | A | B |  |  |
| AC | 0.000 |  | B |  |  |
| SB | 0.000 |  | B |  |  |
|  |  |  |  |  |  |
| **Category** | ***Subdoligranulum*** | **Groups** | |  |  |
| SB | 0.579 | A |  |  |  |
| Control | 0.309 | A | B |  |  |
| AC | 0.222 | A | B |  |  |
| SB plus AC | 0.105 |  | B |  |  |
|  |  |  |  |  |  |
| **Category** | ***Acidaminococcus*** | **Groups** | |  |  |
| Control | 0.451 | A |  |  |  |
| SB | 0.252 | A | B |  |  |
| AC | 0.120 | A | B |  |  |
| SB plus AC | 0.056 |  | B |  |  |
|  |  |  |  |  |  |
| **Category** | ***Dorea*** | **Groups** | |  |  |
| Control | 0.357 | A |  |  |  |
| SB | 0.295 | A |  |  |  |
| AC | 0.112 |  | B |  |  |
| SB plus AC | 0.067 |  | B |  |  |
|  |  |  |  |  |  |
| **Category** | ***Cupriavidus*** | **Groups** | |  |  |
| SB | 0.372 | A |  |  |  |
| Control | 0.184 | A | B |  |  |
| AC | 0.009 |  | B |  |  |
| SB plus AC | 0.006 |  | B |  |  |
|  |  |  |  |  |  |
| **Category** | ***Streptococcus*** | **Groups** | |  |  |
| Control | 0.222 | A |  |  |  |
| SB plus AC | 0.064 | A | B |  |  |
| SB | 0.053 |  | B |  |  |
| AC | 0.041 |  | B |  |  |
|  |  |  |  |  |  |
| **Category** | ***Pseudobutyrivibrio*** | **Groups** | |  |  |
| SB | 0.187 | A |  |  |  |
| Control | 0.124 | A | B |  |  |
| AC | 0.064 |  | B |  |  |
| SB plus AC | 0.027 |  | B |  |  |
|  |  |  |  |  |  |
| **Category** | ***Propionibacterium*** | **Groups** | |  |  |
| SB plus AC | 0.339 | A |  |  |  |
| Control | 0.068 |  | B |  |  |
| SB | 0.040 |  | B |  |  |
| AC | 0.000 |  | B |  |  |
|  |  |  |  |  |  |
| **Category** | ***Odoribacter*** | **Groups** | |  |  |
| SB | 0.095 | A |  |  |  |
| Control | 0.094 | A |  |  |  |
| AC | 0.035 | A | B |  |  |
| SB plus AC | 0.018 |  | B |  |  |
|  |  |  |  |  |  |
| **Category** | ***Lachnobacterium*** | **Groups** | | |  |
| SB | 0.081 | A |  |  |  |
| Control | 0.060 | A | B |  |  |
| AC | 0.014 |  | B | C |  |
| SB plus AC | 0.007 |  |  | C |  |
|  |  |  |  |  |  |
| **Category** | ***Escherichia_Shigella*** | **Groups** | |  |  |
| AC | 0.159 | A |  |  |  |
| SB plus AC | 0.037 | A | B |  |  |
| SB | 0.001 | A | B |  |  |
| Control | 0.001 |  | B |  |  |
|  |  |  |  |  |  |
| **Category** | ***Anaerostipes*** | **Groups** | |  |  |
| SB | 0.068 | A |  |  |  |
| Control | 0.042 | A | B |  |  |
| SB plus AC | 0.022 |  | B |  |  |
| AC | 0.017 |  | B |  |  |

**Table S6: Genera which are significantly different between groups after treatments.** Groups that share a common letter are not significantly different while groups that do not share a letter (A or B or C) are significantly different (P<0.05 for each). SB = *Saccharomyces boulardii* CNCM I-745, AC = Amoxicillin-Clavulanate.

| **Category** | ***Ralstonia*** | **Groups** | |
| --- | --- | --- | --- |
| SB plus AC | 13.043 | A |  |
| SB | 9.942 | A | B |
| Control | 6.570 | A | B |
| AC | 0.003 |  | B |
|  |  |  |  |
| **Category** | ***Bacteroides*** | **Groups** | |
| SB plus AC | 39.342 | A |  |
| AC | 33.320 | A | B |
| Control | 21.997 |  | B |
| SB | 20.311 |  | B |
|  |  |  |  |
| **Category** | ***Roseburia*** | **Groups** | |
| Control | 19.333 | A |  |
| SB | 17.244 | A | B |
| AC | 14.604 | A | B |
| SB plus AC | 10.143 |  | B |
|  |  |  |  |
| **Category** | ***Bradyrhizobium*** | **Groups** | |
| SB plus AC | 0.590 | A |  |
| Control | 0.173 | A | B |
| SB | 0.171 | A | B |
| AC | 0.004 |  | B |
|  |  |  |  |
| **Category** | ***Lachnospira*** | **Groups** | |
| Control | 2.459 | A |  |
| AC | 1.994 | A | B |
| SB | 1.810 | A | B |
| SB plus AC | 0.239 |  | B |
|  |  |  |  |
| **Category** | ***Alistipes*** | **Groups** | |
| Control | 2.238 | A |  |
| SB | 0.970 |  | B |
| SB plus AC | 0.858 |  | B |
| AC | 0.632 |  | B |
|  |  |  |  |
| **Category** | ***Propionibacterium*** | **Groups** | |
| SB plus AC | 0.327 | A |  |
| Control | 0.068 |  | B |
| SB | 0.052 |  | B |
| AC | 0.000 |  | B |
|  |  |  |  |
| **Category** | ***Stenotrophomonas*** | **Groups** |  |
| SB plus AC | 0.148 | A |  |
| SB | 0.011 |  | B |
| Control | 0.001 |  | B |
| AC | 0.000 |  | B |
|  |  |  |  |
| **Category** | ***Parabacteroides*** | **Groups** |  |
| AC | 8.127 | A |  |
| SB plus AC | 2.973 | A | B |
| Control | 2.638 | A | B |
| SB | 1.834 |  | B |
|  |  |  |  |
| **Category** | ***Oscillibacter*** | **Groups** |  |
| SB | 3.550 | A |  |
| AC | 1.332 |  | B |
| Control | 1.060 |  | B |
| SB plus AC | 0.602 |  | B |
|  |  |  |  |
| **Category** | ***Sutterella*** | **Groups** |  |
| SB | 1.374 | A |  |
| AC | 1.224 | A | B |
| SB plus AC | 0.691 | A | B |
| Control | 0.435 |  | B |
|  |  |  |  |
| **Category** | ***Catenibacterium*** | **Groups** |  |
| Control | 1.818 | A |  |
| SB plus AC | 0.846 | A | B |
| AC | 0.001 |  | B |
| SB | 0.000 |  | B |
|  |  |  |  |
| **Category** | ***Dorea*** | **Groups** | |
| Control | 0.357 | A |  |
| SB | 0.318 | A | B |
| AC | 0.188 | A | B |
| SB plus AC | 0.167 |  | B |
|  |  |  |  |
| **Category** | ***Cetobacterium*** | **Groups** | |
| SB plus AC | 0.369 | A |  |
| Control | 0.000 |  | B |
| AC | 0.000 |  | B |
| SB | 0.000 |  | B |
|  |  |  |  |
| **Category** | ***Pseudobutyrivibrio*** | **Groups** | |
| SB | 0.256 | A |  |
| AC | 0.126 | A | B |
| Control | 0.124 | A | B |
| SB plus AC | 0.095 |  | B |
|  |  |  |  |
| **Category** | ***Cupriavidus*** | **Groups** |  |
| SB | 0.247 | A |  |
| Control | 0.184 | A | B |
| AC | 0.020 |  | B |
| SB plus AC | 0.010 |  | B |
|  |  |  |  |
| **Category** | ***Oscillospira*** | **Groups** | |
| SB | 0.126 | A |  |
| Control | 0.066 | A | B |
| AC | 0.052 | A | B |
| SB plus AC | 0.026 |  | B |
|  |  |  |  |
| **Category** | ***Anaerostipes*** | **Groups** | |
| SB | 0.065 | A |  |
| AC | 0.047 | A | B |
| Control | 0.042 | A | B |
| SB plus AC | 0.015 |  | B |
|  |  |  |  |
| **Category** | ***Megasphaera*** | **Groups** | |
| Control | 0.149 | A |  |
| AC | 0.039 | A | B |
| SB plus AC | 0.021 | A | B |
| SB | 0.001 |  | B |
|  |  |  |  |
| **Category** | ***Xylanibacter*** | **Groups** | |
| Control | 0.117 | A |  |
| SB | 0.001 |  | B |
| AC | 0.001 |  | B |
| SB plus AC | 0.000 |  | B |
|  |  |  |  |
| **Category** | ***Anaerobiospirillum*** | **Groups** | |
| SB plus AC | 0.015 | A |  |
| AC | 0.000 |  | B |
| Control | 0.000 |  | B |
| SB | 0.000 |  | B |
|  |  |  |  |
| **Category** | ***Bulleidia*** | **Groups** | |
| SB plus AC | 0.046 | A |  |
| Control | 0.004 | A | B |
| AC | 0.000 |  | B |
| SB | 0.000 |  | B |

**Table S7: Restricted Foods List**

| Yogurt with active cultures |
| --- |
| Fermented milk products (yogurt, sour cream, aged cheeses) |
| Butter milk |
| Kefir |
| *S boulardii* mixtures |
| Sauerkraut (not pasteurized) |
| Naturally fermented vegetables |
| Fermented soybean and soybean paste (tempeh, miso, natto) |
| Kimchi (Korean fermented cabbage with garlic and hot peppers) |

**Table S8. Gastrointestinal Symptom Rating Scale (GSRS)**

|  | | | | | | |  |  |
| --- | --- | --- | --- | --- | --- | --- | --- | --- |
| (1) | | No discomfort at all. | (4) | Moderately severe discomfort. | | |  |  |
| (2) | | Slight discomfort. | (5) | Severe discomfort. | | |  |  |
| (3) | | Mild discomfort. | (6) | Very severe discomfort. | | |  |  |
|  | |  |  |  | | |  |  |
| **GSRS items** | | | | | | | **Response**  **(1 to 6)** | |
| 1 | | Have you been bothered by stomach ache or pain during the past week? (Stomachache refers to all kinds of aches or pains in your stomach or belly.) | | | | |  | |
| 2 | | Have you been bothered by heartburn during the past week? (By heartburn we mean a burning pain or discomfort behind the breastbone in your chest.) | | | | |  | |
| 3 | | Have you been bothered by acid reflux during the past week? (By acid reflux we mean regurgitation or flow of sour or bitter fluid into your mouth) | | | | |  | |
| 4 | | Have you been bothered by hunger pains in the stomach or belly during the past week? (This hollow feeling in the stomach is associated with the need to eat between meals.) | | | | |  | |
| 5 | | Have you been bothered by nausea during the past week? (By nausea we mean a feeling of wanting to be sick.) | | | | |  | |
| 6 | | Have you been bothered by rumbling in your stomach or belly during the past week? (Rumbling refers to vibrations or noise in the stomach) | | | | |  | |
| 7 | | Has your stomach felt bloated during the past week? (Feeling bloated refers to swelling in the stomach or belly.) | | | | |  | |
| 8 | | Have you been bothered by burping during the past week? (Burping refers to bringing up air or gas through the mouth.) | | | | |  | |
| 9 | | Have you been bothered by passing gas or flatus during the past week? (Passing gas or flatus refers to the release of air or gas from the bowel.) | | | | |  | |
| 10 | | Have you been bothered by constipation during the past week? (Constipation refers to a reduced ability to empty the bowels.) | | | | |  | |
| 11 | | Have you been bothered by diarrhea during the past week? (Diarrhea refers to frequent loose or watery stools.) | | | | |  | |
| 12 | | Have you ever been bothered by loose stools during the past week? (If your stools have been alternately hard and loose, this question only refers to the extent you have been bothered by the stools being loose.) | | | | |  | |
| 13 | | Have you been bothered by hard stools during the past week? (If your stools have been alternately hard and loose, this question only refers to the extent you have been bothered by the stools being hard.) | | | | |  | |
| 14 | | Have you been bothered by an urgent need to have a bowel movement during the past week? (This urgent need to open your bowels makes you rush to the toilet.) | | | | |  | |
| 15 | | When going to the toilet during the past week, have you had a feeling or not completely emptying your bowels? (The feeling that after finishing a bowel movement there is still more stool that needs to be passed.) | | | | |  | |
